# The Effect of Exogenous Amylase Supplementation on the Nutritional Value of Pea (*Pisum sativum* L.) for Broiler Chickens

**DOI:** 10.3390/ani13050816

**Published:** 2023-02-23

**Authors:** Katarzyna Perz, Sebastian Andrzej Kaczmarek, Sebastian Nowaczewski, Aaron Joell Cowieson, Marcin Hejdysz

**Affiliations:** 1 Department of Animal Breeding and Product Quality Assessment, Poznan University of Life Sciences, Słoneczna 1, 62-002 Suchy Las, Poland; 2 Department of Animal Nutrition, Poznan University of Life Sciences, Wołyńska 33, 60-637 Poznan, Poland; 3 DSM Nutritional Products, 4303 Kaiseraugst, Switzerland

**Keywords:** pea, amylase, nutritional value, digestibility, poultry

## Abstract

**Simple Summary:**

Pea (*Pisum sativum* L.) can be successfully grown and harvested in almost all the climatic zones across the world. Currently, the main protein source that is used in animal nutrition is soybean meal (SBM). Pea seeds are characterized by a relatively high content of crude protein and starch; therefore, they can serve as a potential energy source when included in feed. However, a portion of starch present in pea seeds is defined as resistant starch (RS). Previous studies have shown that the extrusion process significantly reduced the content of RS; therefore, enzyme supplementation may be more economically advantageous because it is known as an efficient method to improve the digestibility of nutrients. Amylase can be highly beneficial for pea seeds due to the presence of a relatively high starch concentration. However, reports regarding the use of amylase alone in diets based on pea seeds are limited. The present study hypothesized that exogenous amylase can exhibit a positive influence on the nutritional value of pea seeds when included in the feed of broiler chickens.

**Abstract:**

The present study aimed to investigate whether the exogenous addition of amylase enhances the nutritional value of pea seeds for broiler chickens. In total, 84 1-day-old male broiler chickens (Ross 308) were used for the experimental study. During the first phase of the experiment (1–16 d), all birds in each treatment were fed with a corn–soybean meal reference diet. After this time, the first treatment (control) was still fed the reference diet. In the second and third treatment, 50% of the reference diet was replaced with 50% pea seeds. In addition, the third treatment was supplemented with exogenous amylase. Animal excreta were collected on 21 d and 22 d of the experiment. The birds were sacrificed at the end of the experiment (23 d), and samples of ileum content were collected. The experimental results showed that the exogenous addition of amylase significantly improved (*p* < 0.05) the apparent ileal digestibility (AID) of the crude protein (CP), starch, and dry matter (DM) of pea. In addition, an improvement in the AID of essential amino acids in pea seeds (except Phe) was observed. The trend in the AME_N_ values was also noted (*p* = 0.076). It can be concluded that supplementation with exogenous amylase improves the nutritional value of pea seeds in broiler chicken nutrition.

## 1. Introduction

Pea (*Pisum sativum* L.) can be successfully grown and harvested in almost all the climatic zones across the world. Currently, the main protein source that is used in animal nutrition is soybean meal (SBM). However, alternative protein sources obtained particularly from non-GMO plants have been the subject of interest for many authors conducting studies in poultry nutrition [1,2,3].

Pea seeds are characterized by a relatively high content of crude protein and starch, and they can serve as a potential energy source when included in feed. It has been reported that starch is the most important energy source for poultry [4], and starch widely determines the apparent metabolizable energy (AME_N_) value. Starch is usually digested by the endogenous enzymes, but the efficiency of digestion depends on many factors, such as growth phase [5], composition of diet, and the chemical structure of starch [6]. A small portion of starch present in pea seeds is defined as resistant starch (RS), which, depending on the subtype, is harder to digest or not digested at all [7].

Recently, several methods have been explored to enhance the nutritional value of pea seed meals. Technological processes are very effective because they induce changes in the molecular structure of starch. Previous studies have shown that the extrusion process significantly reduced the content of RS in pea seeds [8], improving the digestibility of starch, CP, and, crude fat in broiler chickens [2]. However, these techniques are expensive and led to increases in the cost of production. Compared with this process, enzyme supplementation may be more economically advantageous. Amylase can be highly beneficial for pea seeds because of the presence of a relatively high starch concentration. However, reports regarding the use of amylase alone in diets based on pea seeds are limited. Earlier studies demonstrated the positive impact of amylase when used in soybean–corn diets. Some authors showed that the addition of amylase improves growth performance [9], the digestibility of starch and CP [9,10], and AME_N_ value [5,11] in broiler chickens. In contrast, Kaczmarek et al. (2014) [12] showed that amylase supplementation in a corn–soybean meal diet did not cause any impact on starch and CP digestibility, or on AME_N_ values. Based on the above-mentioned results and due to the lack of information about the effects of amylase supplementation when added to a diet based on pea, the present study hypothesized that exogenous amylase could exhibit a positive influence on the nutritional value of pea seeds when included in the feed of broiler chicken. Moreover, the amount of excreted sialic acid will allow for the estimation of endogenous losses [13] after amylase supplementation.

The aim of the present study was to determine the influence of amylase on nutrient digestibility, energy value, and the excretion of sialic acid in pea-seed-based diets for broiler chickens.

## 2. Materials and Methods

All animal procedures were conducted in accordance with the guidelines proposed by the Polish Council of Animal Care [14].

Pea seeds

The samples of pea seeds (*Pisum sativum L.*) cv. Mentor were obtained from the Polish Plant Breeding Station located in Tulce, and harvested in 2019 in Poland. The seeds were initially ground by using a hammer mill (RG11 model; Zuptor, Gostyn, Poland) with a screen size of 2.0 mm. The chemical composition and the antinutritional factors regarding the concentration of pea are shown in Table 1.

### 2.1. Bird Management

This experiment was conducted in a completely randomized design with a control group and two experimental groups, which differed by the addition of exogenous α-amylase. The study was conducted on 84 1-day-old male broiler chickens (Ross 308) with an initial individual weight of 42 ± 2 g, which were obtained from a Polish hatchery (Dan Hutch, Wolsztyn, Poland). Each group was composed of 14 replicates, with 2 birds in each replicate. All the environmental conditions were controlled and kept constant and were in agreement with the recommendations of AVIAGEN [15]. During the first 7 days of the experiment, the temperature was maintained at about 33 °C and then gradually reduced to 23 °C. The chickens were exposed to light for 24 h during the first 7 days, and then the time of light exposure was gradually reduced to 16 h. Birds were kept in metabolic cages in one common room, and collection trays were installed in every cage. Access to the feed and water was ad libitum during the entire experiment.

### 2.2. Diets

All diets were prepared according to AVIAGEN recommendations [15], such that they meet the nutritional needs of the chickens. The chemical composition and the nutritional value of the reference diet (control) are presented in Table 2. Birds in treatment 1 (control) were fed with a reference diet from 1 d to 16 d of the experiment. Birds in treatment 2 and treatment 3 were fed with the same reference diet from 1 d to 16 d of the experiment, and after this period, 50% of the reference diet was replaced with pea seed. The third treatment was supplemented with exogenous amylase, according to the dosage recommended by the manufacturer of the enzyme (0.14 g/kg; 80 KNU/kg as fed). The enzyme was obtained from a commercial source (Rononzyme HiStarch; DSM Nutrition, Heerlen, The Netherlands). Titanium dioxide (3 g/kg as fed) was added to each diet in each treatment, which serves as a nonabsorbable marker to determine the digestibility coefficients and AME_N_ value.

### 2.3. Samples Collection

For excreta collection, collection trays were installed under each cage, and excreta were collected thrice a day at 21 d and 22 d of experiment. One sample was collected from each replicate group from each treatment. Hence, the total number of samples collected was 14. At the end of the experiment (23 d), all the birds were slaughtered by cervical dislocation. The ileum was removed and the ileal digesta (15 cm, adjacent to the ileocecal junction) was acquired as samples for further analysis. To ensure that a sufficient amount of material was available for chemical analysis, digesta samples of two birds were combined to form a single sample, and thus the total number of samples obtained was 14. Immediately after sample collection, each sample was frozen and then lyophilized.

### 2.4. Chemical Analysis

All samples were ground and passed through a 0.5 mm sieve. Chemical analysis of pea seeds (ran in duplicate) was carried out to determine the amounts of DM, CP, ether extract, crude ash, neutral detergent fiber (NDF), aNDF (NDF with heat-stable amylase and expressed inclusive of residual ash), and acid detergent fiber (ADF; expressed inclusive of residual ash), which was performed according to AOAC standard methods 934.01, 976.05, 920.39, 984.27, 973.18, and, 942.05, respectively [16]. These methods were used to determine the content of DM and CP in digesta and excreta. The assay kit (Megazyme International, Dublin, Ireland) was prepared using thermostable α-amylase and amyloglucosidase according to the standard guidelines of AOAC [16] using method 996.11. This method was used to determine the starch content in pea seeds, diet formulations, and digesta. RS assay kit (Megazyme International, Wicklow, Ireland) was prepared according to the method 2002.02 [16], with a slight modification in the time of incubation, as presented by Weurding et al. (2001) [17], and was used for the determination of the content of RS in pea seeds. Gross energy was calculated in pea seeds, feed, and excreta by using an adiabatic bomb calorimeter (KL 12 Mn; Precyzja-Bit PPHU, Bydgoszcz, Poland) standardized with benzoic acid. The tannins were evaluated according to the method described by Kuhla and Ebmeier (1981) [18]. Phytates were determined according to the method presented by Haug and Lantzsch (1983) [19]. RFOs were determined using a high-resolution gas chromatography technique according to the method suggested by Zalewski et al. (2001) [20]. Phosphorus was determined using the colorimetric method with ammonium molybdate, and calcium by the spectrophotometric method using a split AES Agilent instrument. Non-starch polysaccharides (NSP) in pea seeds were determined by using gas–liquid chromatography-neutral sugars, according to method presented by Englyst and Cummings (1988) [21], with modifications presented by Slominski and Campbell (1990) [22]. Colorimetry method was used for uronic acids, according to method presented by Scott (1979) [23]. Postcolumn derivatization using ninhydrin reagent (according to method 994.12) [16] was performed on an AAA-400 Automatic Amino Acid Analyzer (INGOS s.r.o., Prague, Czech Republic) to determine the amino acid content in pea seeds, experimental diets, and digesta samples. The concentrations of titanium dioxide in diet formulations, digesta, and excreta samples were determined by using the method described by Short et al. (1996) [24], and each sample was prepared according to the procedure presented by Myers et al. (2004) [25]. The excreted free and total sialic acid content was evaluated according to the method presented by Jourdian et al. (1971) [26] after extracting crude mucins from excreta [27]. The determination of WEV was carried out as follows: 1 g of sample was mixed with 5 mL distilled water for 1 h at 40 °C and was centrifuged at 10,000 g for 10 min at 4 °C. The supernatant was withdrawn and viscosity was determined in a Brookfield Digital DV-II+ cone/plate viscometer (Brookfield Engineering Laboratories Inc., Stoughton, MA, USA) maintained at 40 °C at a shear rate of 12 × s^−1^. WEV units are mPas·s = cP = 1 × 100 dyne s cm^2^.

### 2.5. Calculations and Statistical Analysis

Apparent ileal digestibility (AID) values in reference diet (treatment 1) for DM, CP, starch, and amino acids were calculated from the ratio of titanium dioxide concentration in the diet to its concentration in digesta (indigestible marker method) by using the following formula (with starch (S) as an example):

$$AID=\left(1-\left(\left(\frac{TiO_{2\;diet\;}}{TiO_{2\;digesta}}\right)\times \left(\frac{S_{digesta}}{S_{diet}}\right)\right)\right)\times 100\%$$

where S represents the content of starch and TiO_2_ is the dietary marker.

The AID values of DM, CP, starch, and amino acids in pea seeds (treatment 2 and 3) were determined (difference method) using the following formula:

$$AID_{Starch}=\frac{\left(AID_{Starch\;diet}\times C_{Starch\;diet}-AID_{Starch\;reference}\times C_{Starch\;reference}\times 0.50\right)}{\left(C_{Starch\;diet}-C_{Starch\;reference}\times 0.50\right)}$$

where AID_Starch_—digestibility of starch; AID_Starch diet_—digestibility coefficient of starch in diet; C_Starch diet_—concentration of starch in diet; 0.50—amount of investigated pea seeds in diet; AID_Starch reference_—digestibility coefficient of starch in reference diet; and C_Starch reference_—concentration of starch in reference diet.

The difference method approach was used to calculate the AME_N_ and apparent ileal digestibility coefficient of various dietary components contained in pea. The AME_N_ value was corrected to zero nitrogen balance using 34.4 kJ/g of N retained [28] and was calculated by using the following equation (treatment 1):

$$AME_{N}=GE_{diet}-\left[GE_{excreta}\times \left(\frac{TiO_{2\;diet}}{TiO_{2\;excreta}}\right)\right]-0.0344\times \left[N_{diet}-\left(N_{excreta}\times \left(\frac{TiO_{2\;diet}}{TiO_{2\;excreta}}\right)\right)\right]$$

where GE represents the gross energy (kcal/kg), N represents nitrogen, and TiO_2_ is the dietary marker.

The AME_N_ value for treatment 2 and 3 was estimated using the formula:

$$AME_{N\;Pea\;seeds}=(AME_{N\;Pea\;diet}-\left[AME_{N\;reference\;diet}\times 0.5\right]/0.5)$$

where 0.50 is the proportion of the investigated pea seeds in the pea diet and 0.50 is the proportion of the reference diet in the pea diet.

The obtained data were explored to discard any possible outliers. The experimental results were analyzed by using statistical software SAS, (2012). Pooled standard error of mean and group mean values were calculated. Significant differences between means values of the factors investigated in this study were estimated by performing a *t*-test at a significance level of *p* ≤ 0.05. The following formula was used:

$$t=\frac{{\bar{Χ}}_{1}-{\bar{Χ}}_{2}}{\sqrt{\frac{s_{1}^{2}}{n_{1}}+}\frac{s_{2}^{2}}{n_{2}}}$$

where *t*-Student’s *t*-test value ${\bar{Χ}}_{1}-{\bar{Χ}}_{2}$ is the difference in the means between the two groups being compared; $s_{1}^{2},s_{2}^{2}$ are variance estimates from each independent group; and $n_{1},n_{2}$ are the respective sample sizes for each independent group. In this study, the standard error of the mean (SEM) was established as a measure of error.

## 3. Results

The overall mortality was found to be low (<2%) and was not attributed to any specific dietary treatment (data not shown). All research procedures were conducted according to EU directives, and the protocol for this study was approved by the Local Ethics and Animal Experimentation Committee at Poznan University of Life Science.

### 3.1. Nutritional Value and Secretion of Sialic Acid

The effect of enzyme addition on the AID values in pea seeds of DM, CP, and starch is presented in Table 3. The addition of exogenous amylase significantly increased the AID value of DM (from 0.691 to 0.730; *p* < 0.05). A similar result was observed for the AID value of CP, from 0.800 to 0.864. Additionally, the AID value of starch was significantly higher from 0.852 to 0.886 (*p* < 0.05). No significant differences in the AME_N_ values were observed between supplemented and non-supplemented diets. However, a tendency was observed (*p* = 0.076). No significant differences in the amount of excreted total and free sialic acids were observed as a result of amylase addition (Table 3).

### 3.2. Amino Acid Digestibility

The effect of amylase supplementation on the AID of amino acids is presented in Table 3. The addition of amylase significantly increased the AID of almost all essential amino acids (except Phe; *p* < 0.05). A maximum improvement in AID (8% increase) was observed for Thr and Ile. Additionally, a high (7.00%) increase was noted for Lys and Val. In the case of the non-essential amino acids of pea seeds, a significant difference was observed only for Pro, an almost 12.00 % improvement (*p* < 0.05).

## 4. Discussion

Pea seeds are considered to be an important source of protein. The CP concentration was found to be in the reported range of about 208.0–276.0 g/kg as fed depending on the cultivar or type of pea (white or colored flower) [29]. The concentration of starch was found to be higher than the value presented by another study [29]. The content of resistant starch was low (136.6 g/kg as fed) when compared with that observed in a previous study [2]. The pea seeds are a good source of essential amino acids, such as Leu, Lys, and Arg [30,31]. The main RFO found in the pea seeds was stachyose, which was also found by Hejdysz et al. (2015) [29]. The total concentration of NSP was much lower than in data presented by others authors [32]. Indeed, the influence of the cultivar should be distinguished among the factors influencing the concentration of nutrients, as well as antinutritional factors.

Amylase is an enzyme that specifically degrades starch, and supplementation with amylase can have a positive impact on the growth performance of broiler chickens [11], which can be attributed to higher AME_N_ value. No significant differences were noted between non-supplemented and supplemented pea seeds for AME_N_ value in the present study. However, there was a tendency (*p* = 0.076), which is likely related to enhanced starch digestibility. The lack of significant difference in AME_N_, and at the same time an important difference in the AID of starch, was interesting. However, we can speculate that RS in the group without the enzyme was fermented in the caeca and, due to this fact, the energy value level was close between groups. It has been earlier confirmed that fiber and CP also contribute to energy value measurements, although the apparent digestibility of energy alone does not provide a complete response regarding the efficacy of enzyme use [10]. In this study, the addition of amylase significantly increased the AID of starch by 8.00% (*p* < 0.05), similar to the results of Gracia et al. (2003) [9] and Amerah et al. (2017) [10]. The crucial factor that determines the level of digestion is the ratio of amylose to amylopectin, and increasing the ratio of these two compounds reduced starch digestibility. Gunawardena et al. (2010) [33] reported that the ratio of amylose to amylopectin in pea seeds was 31:69, due to which pea native starch is classified as type C. C-type starches do not undergo hydrolysis easily unlike A-type starch, which is found in corn. Since type-A starch is more susceptible to degradation, greater digestibility of this compound is observed in corn-based diets. In addition, a previous study by Amerah et al. (2017) [10] reported that exogenous amylase increased starch digestibility by about 1.12%, while an improvement of 8.00% was noted in this study. However, the level of starch digestibility was found to be much higher in the above-mentioned study. Therefore, it may be concluded that the overall starch digestibility will always be higher with type-A starch. However, it should be noted that the addition of exogenous amylase could also provide good results with type-C starch. Additionally, since exogenous amylase was able to degrade RS effectively, a higher digestibility was observed. On the other hand, Stefanello et al. (2015) [11] found the positive effect of amylase supplementation on the AME_N_ value in conventional diets. However, Kaczmarek et al. (2014) [12] and Amerah et al. (2017) [10] did not observe any increase in the AME_N_ value when amylase was supplemented in conventional diets. It can be assumed that many factors could affect the AME_N_ value, such as the source and the type of starch present in the feed, the different components used in the diet, and the techniques used for the processing of raw materials. However, the crucial factors that determine the AME_N_ value could be the concentration of ANF and RS.

The addition of exogenous amylase significantly increased (*p* < 0.05) the AID of DM and CP by about 5.64% and 3.99% in pea seeds, respectively. An improvement in the AID of DM is the result of an overall improvement in the AID of starch and CP. Gracia et al. (2003) [9] and Amerah et al. (2017) [10] also reported an improvement in the digestibility of CP in studies where conventional diets based on corn and SBM were used. Starch granules contain almost 3 g of protein per 1 kg of starch [7]. As a result of the hydrolysis of starch glycosidic bonds, the portion of protein molecules that were involved in the formation of complexes with starch are now be available for further digestion processes. Another factor that may contribute to improvements in the AID of CP is the fact that the addition of amylase could, by increasing the digestibility of starch and RS, have an effect on the viscosity of the digesta. This could have resulted in changes in the passage time of digesta through the gastrointestinal tract, thus prolonging the time for the action of endogenous proteases. An increase in the concentration of exogenous amylase in diets used for chickens resulted in the lower production and activity of endogenous amylase [34]. In addition, Jiang et al. (2008) [35] showed that amylase supplementation reduces the mRNA expression of amylase. The results of the present study indicate an increasing in the AID of almost all essential amino acids (except Phe). We can assume that a reduction in the production of endogenous amylase can be attributed to the enhanced AID of amino acids. The probable mechanism can be due to the reduced utilization of amino acids for the production of amylase by the pancreas. However, this finding should be confirmed in future studies. As mentioned earlier, starch binds to specific domains of the amylase enzyme. The cleavage of glycosidic bonds in the presence of amylase increases the availability of the substrate to enzymatic hydrolysis by proteases, which further leads to an increase in the digestibility of amino acids. Liu et al. (2020) [36] reported that increasing the concentration of RS decreases the digestibility of CP. Amylase supplementation could effectively decrease the levels of RS in the feed. Schramm et al. (2021) [37] reported that amylase addition increased the digestibility of RS. Therefore, we can conclude that a reduction in the levels of RS resulted in an increase in the digestibility of CP and amino acids.

A previous study indicated that sialic acid excretion was lower after the extrusion process, which can be associated with lower levels of RS [8]. Additionally, sialic acid can be considered to be an indicator of endogenous losses [13]. However, no effect of amylase supplementation on sialic acid secretion was observed in the present study, which indicates that ANF concentration, rather than RS concentration, is a strong determiner of this parameter; however, this requires confirmation.

In conclusion, the addition of exogenous amylase enhances the apparent ileal digestibility of DM, starch, and CP in pea seeds. In addition, the significantly higher apparent ileal digestibility of almost all essential amino acids was observed. The tendency to obtain a higher AME_N_ value was also observed. Therefore, exogenous amylase can improve the nutritional value of pea and affects the nutrients and amino acid utilization in broiler chicken nutrition.

## Figures and Tables

**Table 1:** Chemical composition, amino acids profile, NSP content, and antinutritional factors ^1^.

	Item	
Content (g/kg as fed)	Dry matter	872.00
	Crude protein	242.80
	Crude fat	9.90
	Ash	29.70
	Ca	2.00
	P	4.80
	Starch	487.00
	ADF	80.30
	NDF	199.10
	Resistant starch	136.60
	WEV (cP)	1.34
Essential amino acids (g/kg as fed)	Lys	16.34
	Thr	9.25
	Ile	10.39
	Val	11.78
	Leu	17.89
	Phe	12.09
	His	6.90
	Arg	22.53
	Gly	11.19
Non-essential amino acids (g/kg as fed)	Tyr	7.41
	Ala	12.36
	Asp	33.17
	Glu	42.81
	Ser	13.06
	Pro	5.58
Non-starch polysaccharides (g/kg as fed)	Total	120.40
	Arabinose	25.90
	Xylose	10.80
	Mannose	0.80
	Galactose	5.10
	Glucose	53.60
	Uronic acids	23.80
Antinutritional factors (g/kg as feed)	RFO	4.50
	Raffinose (% of RFO)	14.47
	Stachyose (% of RFO)	44.86
	Verbascose (% of RFO)	40.67
	Phytic P	2.50
	Tannins	0.08

^1^ Each value represents mean of 2 replicates. WEV = water extract viscosity. RFO = raffinose family oligosaccharides.

**Table 2:** The composition and nutritional value of reference diet.

Components	(g/kg as Fed)
Corn	600.00
Soybean meal	292.60
Soybean oil	41.60
Fish meal	29.40
Monocalcium phosphate	10.30
Limestone (<2 mm)	5.10
Premix–broiler ^1^	10.00
Salt	2.90
Sodium bicarbonate	0.10
D, L-Methionine	2.70
L–Lysine-HCl	1.70
L-Threonine	0.60
TiO_2_	3.00
Determined nutritional value (g/kg as fed)	
Metabolizable energy (kcal/kg)	3011.85
Dry matter	882 (0.70) ^2^
Starch	423 (0.89)
Crude protein	212 (0.90)
Amino acids	
Lys	12.80 (0.72)
Thr	8.73 (0.64)
Val	9.00 (0.71)
Ile	8.94 (0.69)
Leu	17.70 (0.73)
Phe	10.80 (0.73)
His	6.80 (0.71)
Arg	15.20 (0.80)
Gly	1.05 (0.67)
Tyr	6.82 (0.70)
Ala	11.00 (0.71)
Asp	22.30 (0.70)
Glu	34.40 (0.80)
Ser	10.50 (0.66)
Pro	13.20 (0.69)
Calculated nutritional value (g/kg as fed)	
Crude fat	72.90
NDF	99.00
ADF	37.30
Ash	28.00
Ca	9.20
P–avail.	4.30
Na	1.60
Cl	3.00

^1^ Provided per kg diet: IU: retinol 11,250, cholecalciferol 2500; mg/kg: tocopherol 80, menadione 2.50, cobalamin 0.02, folic acid 1.17, choline 379, D-pantothenic acid 12.5, riboflavin 7.0, niacin 41.67, thiamin 2.17, D-biotin 0.18, pyridoxine 4.0, ethoxyquin 0.09, Mn 73, Zn 55, Fe 45, Cu 20, I 0.62, Se 0.3, salinomycin 60. ^2^ Apparent ileal digestibility of dry matter and ingredient in reference diet.

**Table 3:** Apparent ileal digestibility (AID) of dry matter, crude protein, starch, amino acids, and AME_N_ (kcal/kg) levels, and the excretion of total and free sialic acid (micromol/g TiO_2_) from broiler chickens fed pea seeds without (−) and with (+) enzyme supplementation ^1^.

Item		−	+	SEM	*p*
AID	Dry matter	0.691	0.730	0.013	0.048
	Crude protein	0.800	0.864	0.020	0.031
	Starch	0.852	0.886	0.011	0.039
	Lys	0.868	0.930	0.013	0.001
	Thr	0.811	0.874	0.019	0.025
	Ile	0.827	0.890	0.015	0.010
	Val	0.832	0.890	0.015	0.012
	Leu	0.835	0.881	0.017	0.012
	Phe	0.839	0.881	0.017	0.107
	His	0.844	0.895	0.012	0.004
	Arg	0.912	0.942	0.008	0.046
	Gly	0.820	0.871	0.015	0.024
	Tyr	0.705	0.742	0.015	0.010
	Ala	0.840	0.886	0.018	0.079
	Asp	0.864	0.889	0.014	0.215
	Glu	0.918	0.940	0.014	0.280
	Ser	0.789	0.829	0.020	0.174
	Pro	0.712	0.795	0.024	0.024
Energy value	AME_N_	2476.83	2579.54	38.215	0.076
Excretion of sialic acid	Free	434.7	431.4	16.9	0.895
	Total	496.2	497.5	18.0	0.959

SEM = Pooled standard error of mean; AMEN = nitrogen corrected metabolizable energy. ^1^ Each value represents mean of 14 replicates.

## Data Availability

Data is available upon request from the corresponding authors.
